# Motion-artifact-free single shot two-beam optical coherence elastography system

**DOI:** 10.1117/1.JBO.29.2.025003

**Published:** 2024-02-22

**Authors:** Asha Parmar, Kanwarpal Singh

**Affiliations:** a Max-Planck-Institute for the Science of Light, Erlangen, Germany; b Max-Planck-Zentrum für Physik und Medizin, Erlangen, Germany; c Friedrich-Alexander-Universität Erlangen-Nürnberg, Department of Experimental Physics, Erlangen, Germany; d McMaster University, Department of Electrical and Computer Engineering, Hamilton, Ontario, Canada

**Keywords:** optical coherence elastography, systemic sclerosis, bulk motion correction

## Abstract

**Significance:**

The assessment of the biomechanical properties of the skin using various imaging techniques has been used as a diagnostic tool in dermatology. Optical coherence elastography (OCE) is one of the techniques that allows for the measurement of elastic properties. OCE relies on measuring tissue displacements induced by external sources. Measuring the tissue’s mechanical properties *in vivo* using OCE is often challenging due to bulk tissue movement.

**Aim:**

This study aimed to develop an OCE system that allows for minimizing the effects of bulk tissue movements. To achieve this, we designed a two-beam OCE system that simultaneously measures the tissue displacement at two locations on the sample. This allows for cancelling the effect of the tissue bulk movement, which is common to both measurement points.

**Approach:**

We used a piezoelectric transducer to generate surface acoustic waves (SAW) in the sample. The velocity of the excited waves, which is proportional to the rigidity of the sample, was measured by calculating the phase delay of the SAW at two locations on the sample. Simultaneous measurement at two locations was achieved by dividing a single light beam into two by focusing on the sample at two different locations. The two beams travel different optical path lengths, and the reflected signals were depth encoded in a single optical coherence tomography scan using a single reference beam.

**Results:**

The system was characterized using different tissue-mimicking phantoms and the skin of healthy volunteers at the wrist and the palm. We achieved an approximately 50-fold increase in phase sensitivity measurement.

**Conclusions:**

We designed a simple two-beam OCE system that effectively minimizes the effect of tissue movement. We believe that the presented system will find immediate applications in the clinic to monitor the progression of systemic sclerosis disease.

## 1 Introduction

Changes in the mechanical properties of the tissue contribute to the development of several different diseases. Elastography can be used as a diagnostic tool for mapping the tissue’s mechanical properties.^1^ The assessment of these properties is very important in diagnosing diseases such as systemic sclerosis at an earlier stage.^2^^–^^4^ Systemic sclerosis is an autoimmune disease that occurs due to the excessive production of collagen in the tissue because of which the skin becomes stiffer and may cause the failure of organs, such as the lungs, liver, heart, and skin.^5^^–^^7^ The gold standard for clinical evaluation of systemic sclerosis is the palpation of skin method, which is highly subjective, so the results can vary dramatically among different evaluators.^8^^–^^10^ Nevertheless, the current procedures still lack sensitivity to quantify the diseases in earlier stages, which is extremely important for reducing the risk of specific complications.^11^^,^^12^ In this direction, optical coherence elastography (OCE) can provide quantifiable measurement of the mechanical properties of the skin with high precision.^1^^,^^5^^,^^6^

Some imaging modalities, such as ultrasound elastography^13^ and magnetic resonance elastography,^14^ have been used to analyze the mechanical properties at the tissue level. At the cellular level, other imaging modalities, such as atomic force microscopy^15^^–^^17^ and traction force microscopy,^18^ have been used. More recently, due to the large penetration depth (up to 2 mm) and spatial resolution (up to 1  μm), optical methods such as OCE have been used for elastography measurements.^19^ OCE measures tissue displacement on the application of an external load. This can be performed in many ways such as contact or non-contact; dynamic, static, or quasi-static; and localized or global.^20^^–^^23^

Dynamic OCE or wave-based OCE, which analyzes the surface acoustic wave (SAW) speed that travels on the surface of the sample, is commonly based on two optical coherence tomography (OCT) imaging modalities, called swept-source OCT (SS-OCT) and spectral-domain OCT (SD-OCT). These modalities offer fast data acquisition and high sensitivity to tissue motion. In OCE, the tissue motion is measured by measuring the change in the relative optical path length between the sample and a reference surface. This is achieved by monitoring the phase of the interference signal from the sample and the reference surface. The phase sensitivity of the system determines the accuracy with which the sample motion can be measured. In SS-OCT, the phase stability of the system suffers from fluctuations in the mechanical movement of the laser sweep, which makes it difficult to achieve a proper synchronization between the data acquisition and the laser sweep and thus high phase stability.^24^ Systems based on the common path SS- OCE approach to increase phase stability have been reported.^25^ Furthermore, SD-OCE schemes also suffer from phase instability due to external sources for instance, fluctuating optical path differences, polarization mismatch, and dispersion mismatch between the sample arm and the reference arm. Also, the common path SD-OCE schemes have been reported to obtain high phase sensitivity down to sub-nanometers.^26^

Until now, all OCE systems have relied on using consecutive A-scans separated in time to measure the propagation of the SAW to determine the elastic properties of the sample.^27^^,^^28^ Common path schemes reduce the system-related phase instability, but even these systems are not free from phase errors that happen between consecutive A-scans specifically due to the tissue bulk movement.^5^ Dual-beam OCT systems have been reported to provide high resolution, higher phase stability, longer imaging depth, and fast imaging speed for tissue imaging.^29^^–^^31^

In this direction, we used a two-beam OCT method, which is novel approach in OCE, to minimize the phase errors related to bulk tissue motion.^24^ We used two beams to simultaneously measure the displacement of the tissue at two different locations on the sample. The simultaneous measurement allows for cancelling the effect of the tissue bulk movement, which is approximately the same at both points. Using this methodology, we achieved a phase stability that is about 50 times higher than a single beam measurement. Using the developed technique, we measured the phase velocity of the SAW and correspondingly the mechanical properties of samples such as agar phantoms and skin. The obtained results for the mechanical properties are comparable to previously reported results.

## 2 Material and Method

### 2.1 System Design

**Fig. 1:**
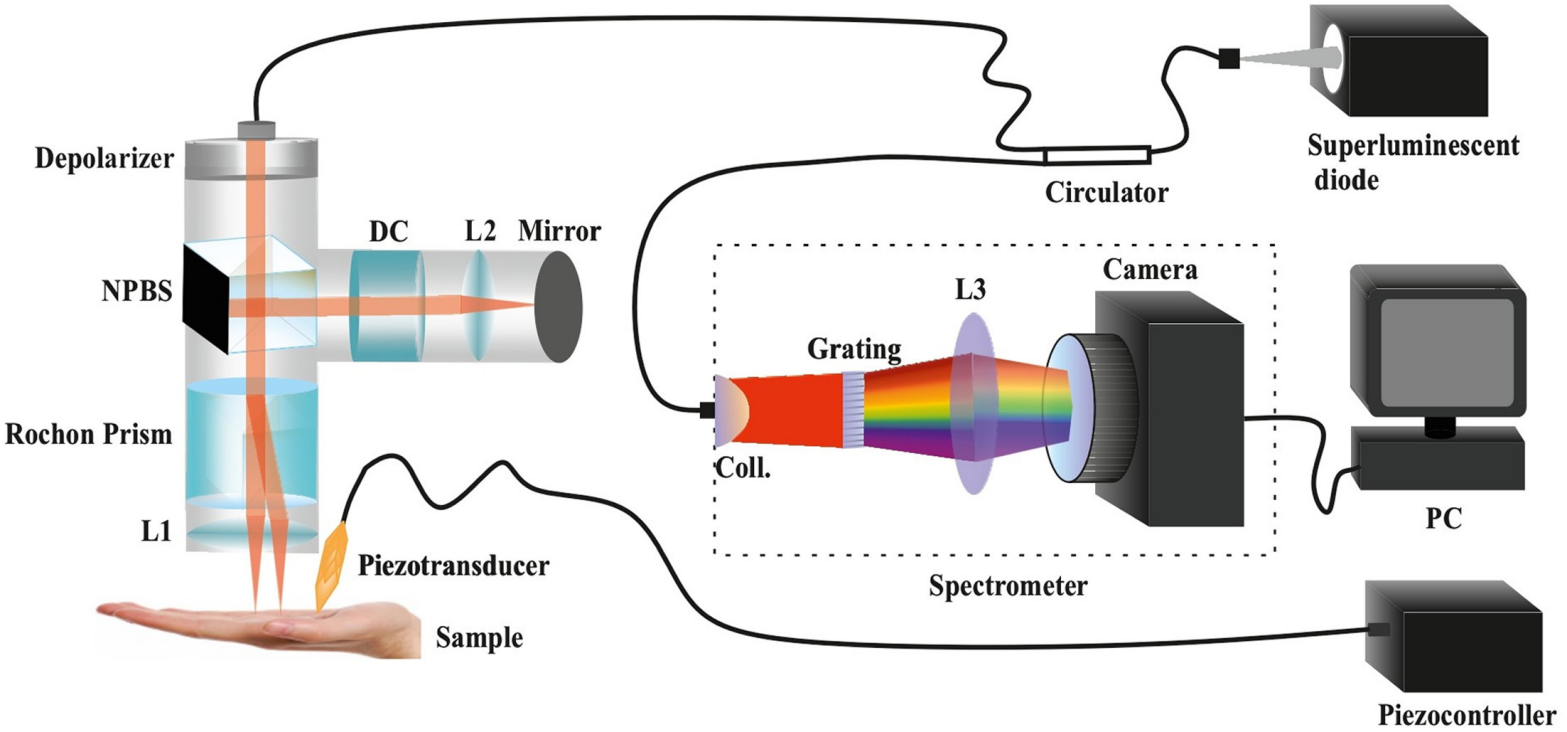
Schematic of two-beam OCE system. Non-polarizing beam splitter (NPBS), L (lens), dispersion compensator (DC), and collimator (Coll.).

The designed SD-OCT system used for the two-beam OCE method is described in our previous work.^32^ In brief, the modified system, shown in Fig. 1, used a super luminescent diode (SLD-371, Superlum) as a light source with a central wavelength of 840 nm and a full-width half maxima bandwidth of 52 nm. The light was coupled to a circulator (850 nm SM Circulator, PM-Optics) and then to a single-mode optical fiber (HI 780, AFW Technologies). The polarized light from the fiber passed through a depolarizer (DPU-25-B, Thorlabs) and split into two parts as the reference signal and the sample signal via a non-polarizing beam splitter (CCM1-BS014/M, Thorlabs). In the sample arm, we used a Rochon prism (RPM10, Thorlabs USA) to divide the sample signal beam into two beams at an angle of 1.5 deg. We used an achromatic lens (AC254-050-B) to focus both beams on the sample. The distance between the focused beams was measured to be 2 mm. The sample was illuminated simultaneously at two different positions. The reflected signals from two different locations of the sample interfered with the reference signal with slightly different optical path lengths.

For dispersion compensation, we used a dispersion compensator (LSM54DC1, Thorlabs) and an Achromatic lens (AC254-050-B) in the reference arm. For the mechanical wave excitation into the sample, we used an electric piezo transducer (PK2FMP1, Thorlabs) at a distance of 3 mm from the first beam and 5 mm from the second beam. The stacked piezo transducer (5.2  mm×7.1  mm×12.6  mm) is capable of generating an 11.2  μm displacement at a voltage of 75 volts. We used a small metallic piece (2  mm×4  mm×2  mm), which was glued on the piezo transducer tip. The piezo transducer was driven at a 0 to 75 V pulsed voltage signal with a 1% duty cycle from a signal generator (National Instruments, United States) at 10 Hz. A TTL signal from the signal generator at 10 Hz was used as a trigger for the OCT frame acquisition. The interfered signal from both arms was detected using a home-built spectrometer. The spectrometer consisted of a 1200 lines/mm diffraction grating (WP-1200/840), an 80 mm focal length lens, and a 2048-pixel line scan camera (RAL2048, Basler AG) operating at 25000-line scans per second. The images were acquired in M-mode and each M-mode scan consisted of 2500 A-scans. The interferograms from the camera were transferred to a host personal computer (NUC10i7FN-Kit- Mini-PC) via an Ethernet cable.

To calculate the tissue displacement (Δx) from the phase difference (ΔΦ) between the reference surface and the sample surface, we used the following equation:

$$\Delta x=\Delta \Phi \frac{\lambda_{0}}{4\pi n},$$ (1)

where $\lambda_{0}$ is the central wavelength of the light and n is the sample refractive index.

For the calculation of the velocity of the induced SAW in the sample, we used the following relation:

$$v=\frac{(x_{1}-x_{2})2\pi f}{\Delta \varphi },$$ (2)

where $\left(x_{1}-x_{2}\right)$ is the distance between the two measurement points, f is the frequency, and Δφ is the phase difference between two SAW measured at two locations. We also calculated the Youngs modulus of the sample using the following relation:

$$E=3.3\rho c_{R}^{2},$$ (3)

where E is Young’s modulus, ρ is the material density ($\approx 1060\;\mathrm{kg}/m^{3}$ for soft tissue),^33^ and $c_{R}$ is the velocity of induced SAW.

### 2.2 Sample Preparation

To test the system, we prepared and measured the elasticity of homogenous tissue-mimicking agar phantoms of 10 mm thickness with different concentrations, i.e., 1%, 2%, and 3%. We also performed measurements on the skin of healthy volunteers at locations such as the palm and wrist. All methods carried out in this work are under relevant guidelines and regulations from the local institutional review board (Ethikkommission der Friedrich-Alexander Universität, Erlangen, Germany). Testing on hand was performed as a self-test on the author of the manuscript who signed informed consent to participate. No other human experiments were performed in this work.

## 3 Results

### 3.1 Phase Sensitivity

**Fig. 2:**
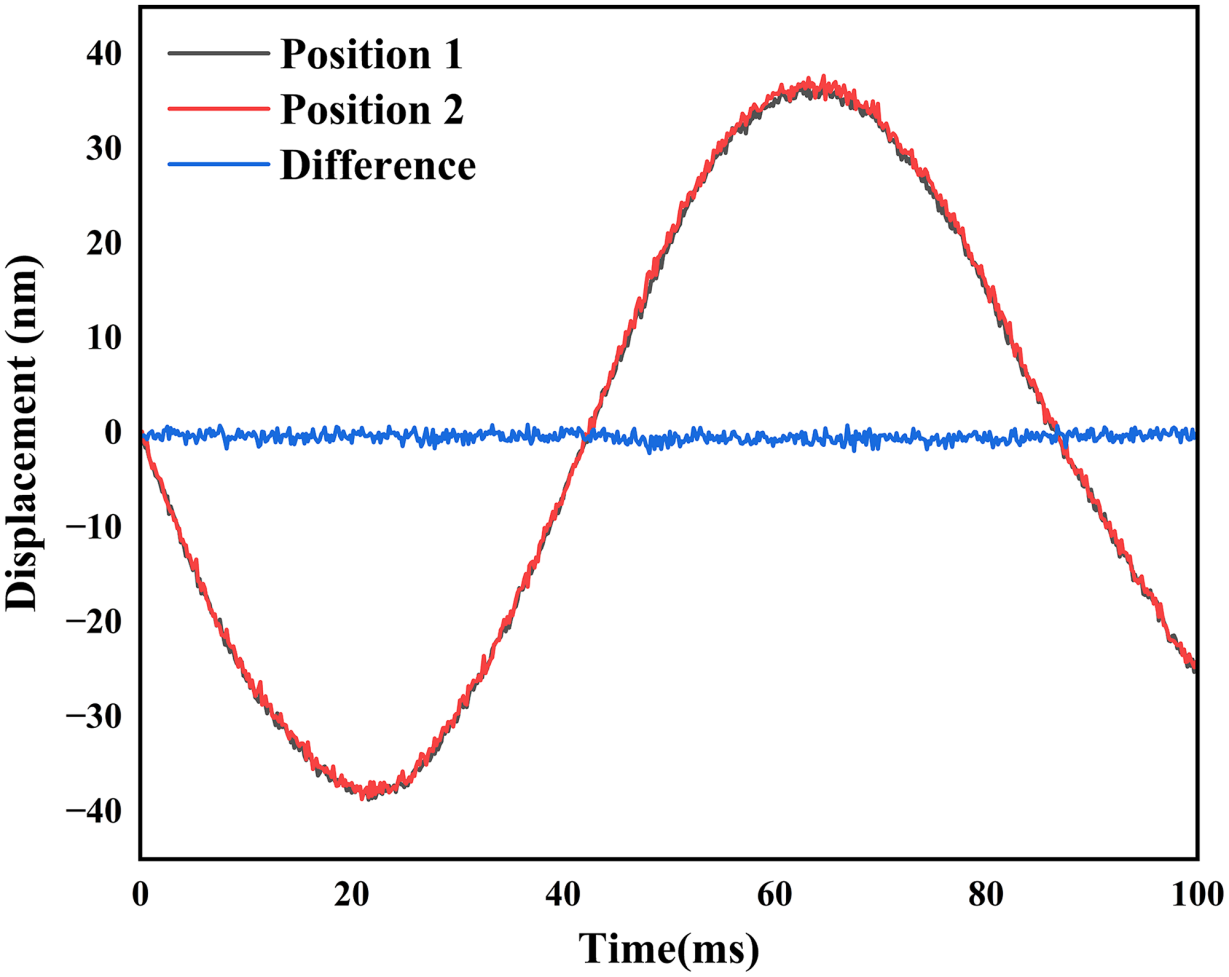
Simultaneously measured displacement at two positions on the glass plate. Also, the difference between the simultaneously measured displacements, which represents the noise of the system in terms of the displacement measurement, is shown.

To measure the phase sensitivity of the two-beam OCE system, we used a glass plate as a sample. We calculated the phase difference of the two beams reflected from the first surface of the glass plate with respect to the reference surface. As we measured the reflected signals from both beams simultaneously, they followed the same phase pattern, which is due to the bulk motion of the glass plate. The measured phase difference for both beams is shown in Fig. 2. To calculate the improvement in the signal-to-noise ratio, we subtracted both signals to get rid of the common noise at both positions. Using Eq. (1), the amplitude of the measured bulk motion between the glass plate and the reference surface was found to be 24.90 nm. After subtracting the common bulk motion, the measured standard deviation of the noise floor was found to be 0.50 nm. This suggests that an ∼50-fold increase in the signal-to-noise ratio can be obtained with a two-beam system compared with the single-point measurement.

### 3.2 Measurement of Amplitude and Phase Velocity of SAW in Agar Phantoms

To further test the system, we measured the amplitude and phase velocity of SAW induced into the agar samples with an electric piezo transducer. The piezo transducer was operated at 10 Hz with a pulsed electric signal at a 1% duty cycle. The velocity and amplitude of the excited SAW were measured on the sample surface with two beams. To measure the phase velocity between the two measuring points, a cross-power spectrum fast Fourier transform (FFT) was performed.^34^ We extracted the phase delay or time delay from the cross-power spectrum FFT. This phase delay was converted to velocity using the known distance between the two-measurement points. We calculated the phase velocity at different frequencies using Eq. (2).

**Fig. 3:**
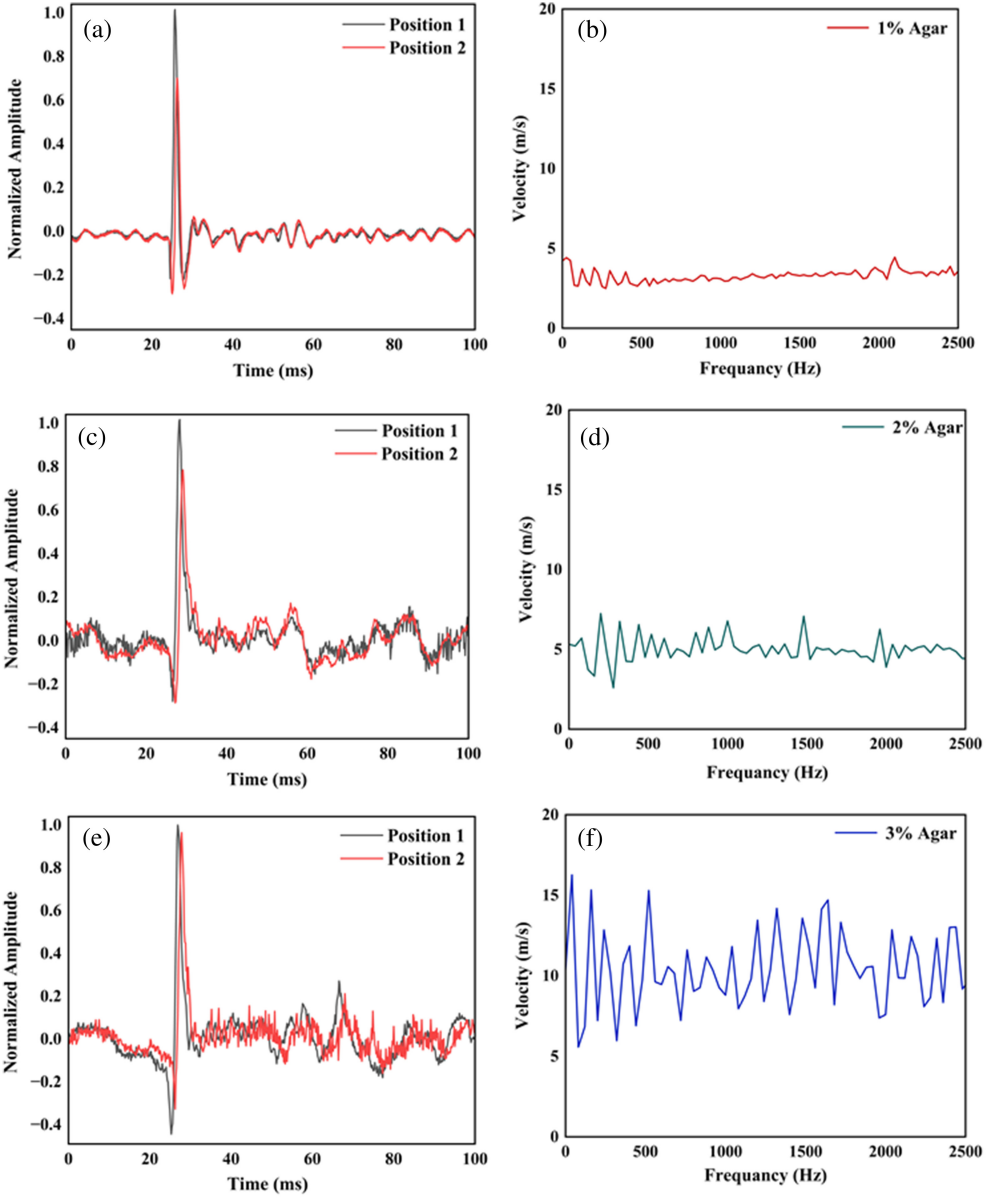
(a), (c), and (e) Measured temporal displacement and (b), (d), and (f) SAW velocity for 1%, 2%, and 3% agar phantoms.

In Fig. 3, we show the normalized amplitude and phase velocity for 1%, 2%, and 3% agar phantoms. The calculated average phase velocity in 1%, 2%, and 3% agar was found to be 3.28±0.37  m/s, 4.98±0.81  m/s, and 10.29±2.4  m/s, respectively. We also calculated the Youngs modulus using Eq. (3) for 1%, 2%, and 3% agar; it was found to be 37.63±.478  kPa, 86.75±2.3  kPa, and 370.38±20.14  kPa, respectively.

### 3.3 Measurement of the Amplitude and Phase Velocity of SAW in Skin

**Fig. 4:**
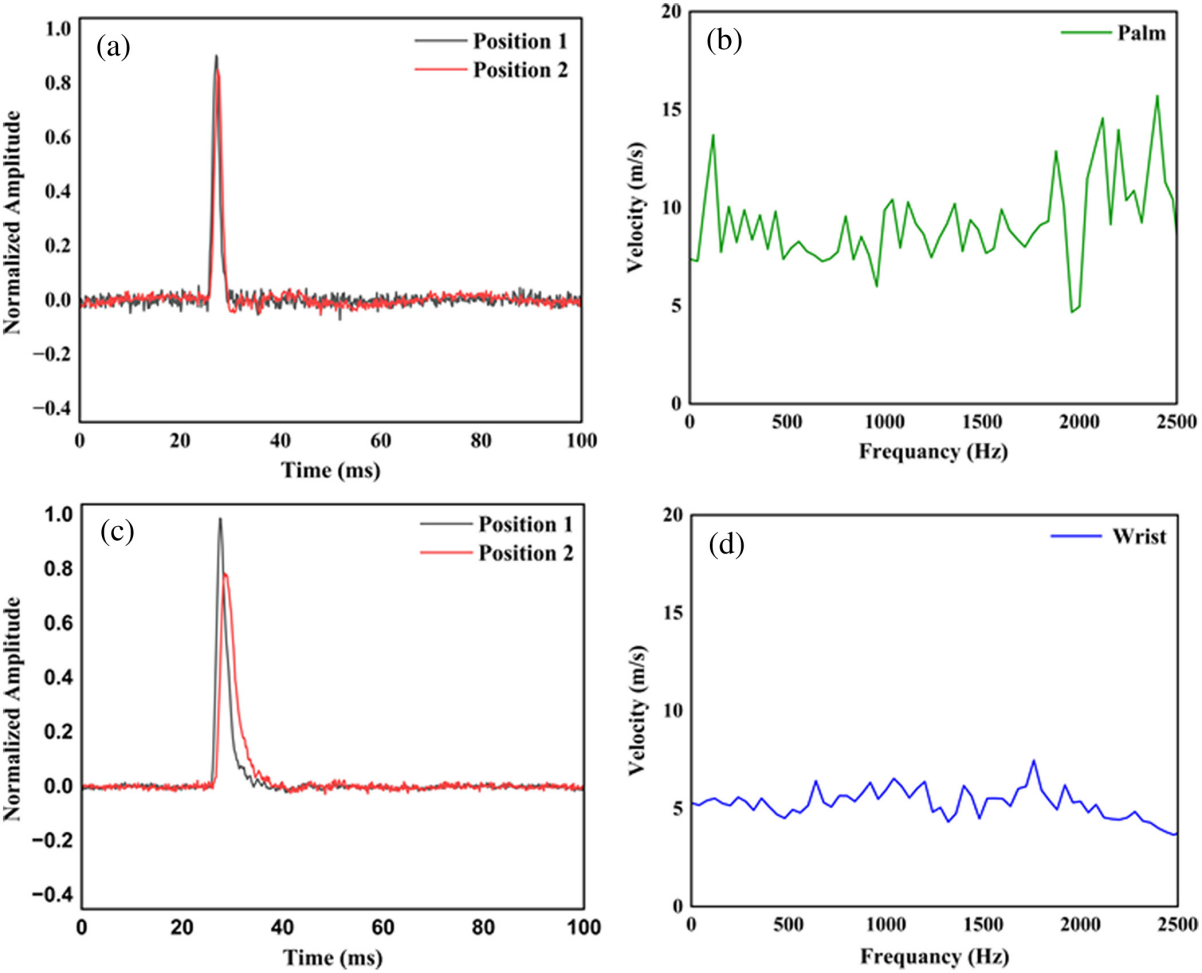
(a) and (c) Measured temporal displacement and SAW velocity (b) and (d) for the skin at the palm and the wrist.

In Fig. 4, we show the normalized amplitude and phase velocity of SAW in the skin at different positions such as palm and wrist. The calculated phase velocity on the palm and wrist was found to be 8.7±2.6  m/s and 5.12±0.68  m/s, respectively. The calculated Young’s modulus of the palm and wrist was 264.76±22.3  kPa and 91.69±1.62  kPa, respectively. The obtained results from phantoms and skin are found to be comparable with previous works.^32^^,^^35^

## 4 Conclusions

We have developed a phase-stable two-beam OCE system to reduce the effect of the bulk tissue moment due to external sources. The tissue bulk moment induces undesired changes in the measured displacement. This situation is unavoidable in clinics, where the bulk tissue movement arises from within the sample itself due to body motion. This issue was resolved by simultaneously measuring the mechanical properties of the tissue at multiple points rather than a single point.^34^^,^^35^ For this, we used an electric piezo transducer and a two-beam OCT method to generate and detect mechanical waves in the tissue. The common phase noise was cancelled out as we focused two beams simultaneously on the sample at two positions. By measuring the displacement between the adjacent A-scans in M mode, we calculated the phase velocity of the tissue caused by the induced waves. The developed two-beam OCE system is capable of reducing phase noise up to 50-fold compared with a single-beam method. We tested this approach using different tissue agar phantoms, which mimic the biomechanical properties of human tissue and the skin. It should be noted that the two-beam configuration can minimize the motion artifacts related to breathing or unwanted body movements, especially when the measurement points are separated by only a few millimeters, but there will always be some tiny tissue displacement that is not common to both the measurement point and hence not fully compensated. Such displacements are much smaller, however, when compared with the tissue displacement due to the traveling SAW, and they affect the measurements minimally. The current designed system is still a contact-based system in which we used an electric piezo transducer for mechanical wave excitation. For faster adaptability in clinical applications, a non-contact-based system would be better suited as one can use a high-frequency ultrasound transducer to induce mechanical waves into the sample. Nevertheless, the current system demonstrates that a two-beam OCE system can be used to effectively reduce the system noise due to bulk tissue movement, and this technique will be helpful in clinical applications of OCE.

## Data Availability

The data that support the findings of this study are available from the corresponding author upon reasonable request.
